# Haemorrhagic bowel syndrome in fattening pigs

**DOI:** 10.1186/s40813-017-0074-1

**Published:** 2017-12-19

**Authors:** Alexander Grahofer, Corinne Gurtner, Heiko Nathues

**Affiliations:** 1 Clinic for Swine, Department of Clinical Veterinary Medicine, Vetsuisse Faculty, University of Bern, Bremgartenstrasse 109a, 3012 Bern, Switzerland; 2 Department of Infectious Diseases and Pathobiology, Institute of Animal Pathology, Vetsuisse Faculty, University of Bern, Länggassstrasse 122, 3012 Bern, Switzerland

**Keywords:** Sudden death, Whey feeding, Mesenteric volvulus, Dysbacteriosis, Antimicrobials

## Abstract

**Background:**

Haemorrhagic bowel syndrome (HBS) is a sporadically occurring disorder in fattening pigs, characterized by sudden death in combination with severe abdominal distension and intense red colouration of the intestine. Deep understanding of aetiology and pathogenesis of HBS are still lacking, although several risk factors are known.

**Case presentation:**

In a continuously stocked fattening farm with 1500 pigs and liquid feeding based on whey, the mortality rate increased from 1.7% to 3.5% during summer time. Sporadic sudden death of growing pigs occurred along with severe abdominal distension as the main sign in these animals. All batches arriving at the farm received in-feed medication with Tiamulin hydrogen fumarate (2 mg/kg body weight/day; according to the license for use in Switzerland) due to detection of *Brachyspira hyodysenteriae* in the past, although a partial sanitation had been conducted thereafter. No changes of the origins, housing and the feeding procedure were reported. A herd examination was conducted revealing a hygiene problem in the feeding system. For further diagnostics a necropsy was performed, showing a pale carcass with a bloated abdomen due to a haemorrhagic infarction of part of the small intestine caused by a mesenteric torsion. Furthermore, a feed analysis was conducted, revealing a pH-value of 5 in the liquid feed, and a severe contamination with *Enterobacteriaceae* was detected. Based on these examinations, HBS was diagnosed. Subsequently, the farmer controlled the pH-value of the liquid feed with formic acid, improved the cleaning procedure of the liquid feeding system and stopped the in-feed medication. Following the implementation of these measures, key performance indicators improved significantly, but 4 months later the same clinical manifestation occurred again. This time huge variations in the pH-value of the liquid feed between different feeding times were recorded and were attributed to improper mixing of the formic acid in the whey tank. After implementation of a technical solution to control the pH-value, the health status improved again.

**Conclusion:**

In the present case, it is likely that the cause of the clinical manifestation of HBS was a contamination of *Enterobacteriaceae* in the liquid feed facilitated by a hygiene problem in the feeding system, and a chronic dysbacteriosis of the intestinal tract due to the non-justified routine use of antimicrobials. Speculatively, the prophylactic antimicrobial treatment was unnecessary and might even have exacerbated the clinical problem.

## Background

The purpose of this report is to describe an outbreak of Haemorrhagic Bowel Syndrome (HBS) with sudden death in a continuously stocked fattening farm with 1500 pigs and liquid feeding based on whey. Furthermore, the batches of pigs arriving at the farm were routinely treated with tiamulin hydrogen fumarate.

HBS was first described 1959 and since then given many different terms, because of diagnostic as well as aetiological confusions about the origin [1, 2]. It is a sporadically occurring disorder in fattening pigs characterized by sudden death in combination with severe abdominal distension and intense red discolouration of the intestinal tract due to a haemorrhagic infarction of the intestine caused by mesenteric volvulus. It primarily affects rapidly growing pigs between four and 6 months of age [1] and mortalities tend to occur in the early hours of the morning accompanied by circulatory disturbances [3]. Deep understanding of the aetiology and pathogenesis of HBS is still lacking [2, 4, 5], but several risk factors have been described. Some authors indicated that HBS could have an infectious aetiology or this condition can be the result of intestinal volvulus of the intestines around the mesentery [1, 3, 5]. With regard to the aetiology of the volvulus, the fermentation of nutrients reaching the large intestine, resulting in caecal distension, is seen as critical. The hypothesis that pigs can die from hypovolemic shock due to excessively high intraabdominal pressure in the absence of intestinal torsion was supported by pressure measurements immediately after death [6]. Furthermore, it is assumed that HBS arises from overgrowth and alterations of bacteria such as *Clostridium perfringens*, *Escherichia coli* and yeast in the intestinal tract producing toxins and damaging compounds. In ruminants various risk factors contributing to bacterial overgrowth have been described [7, 8]. According to a report from South Africa *Clostridium perfringens* was isolated from 40% of submitted intestinal mucosal scrapings of grower pigs with HBS [3]. Furthermore, higher rates of death due to HBS have been described during summer and in conjunction with a highly fermentable liquid feeding [1, 5], possibly due to higher loads of these bacteria. However, cases of HBS also occur during the winter season and/or when a dry feeding system is in use [9]. The epidemio-clinical approach plays a major role in the diagnostic procedure and other causes of sudden death have to be ruled out [2]. Pathological examination allows visualization of intestinal and organ topography and allows the diagnosis of HBS to be made. Identifiable volvulus of the intestine has been a variable finding in necropsies of diseased animals, but sometimes is not seen, probably due to transport or post mortal repositioning of the gut [1, 5]. However, the haemorrhagic infarction und therefore the red discoloration of the gut is still visible even if the volvulus has been repositioned.

Regardless of treatment HBS is a fatal disease. Further research is necessary to investigate management factors that can act as a trigger, and to fully understand any predisposing factors to avoid huge economic losses and improve animal welfare in fattening farms [1–3, 5, 9].

## Case presentation

The case described here occurred in a fattening farm with 1500 Large White- Landrace crossbred pigs, located in the central part of Switzerland. Every week a new batch of approximately 125 growing pigs, vaccinated against Porcine Circovirus Type 2, weighing between 17 and 32 kg and originating from two different breeding farms arrived at the single premises of the farm. When entering, growers were sorted by weight and subsequently housed in groups of 25 pigs per pen until slaughter. Pigs were fed four times a day with liquid feed, which consisted of commercial feed, stabilized whey and water. No irregularities within the timing of the feeding have occurred, because of the employment of an automatic feeding system in the farm. Two different kinds of feed, one for starting and one for finishing, were used on the farm. The starting feed differed in the content of protein and the content of fat from the finishing feed. More information about the feed components and feed content can be found in Tables 1 and 2.

**Table 1:** Feeding characteristics of the starting and finishing diet on the farm

Parameter	value	starting feed	finishing feed
DE	(MJ/kg)	13.50	13.50
CP	(g/kg)	164.24	159.23
CF	(g/kg)	35.26	32.76
Fat	(g/kg)	33.68	28.66
DM	(%)	88.42	88.42
Lys	(g/kg)	11.86	10.65
CM	(g/kg)	6.88	6.42
Thr	(g/kg)	7.54	6.99
Try	(g/kg)	1.92	1.87

*Abbreviation*: *DE* digestible energy, *CP* crude protein, *CF* crude fibre, *DM* dry matter, *Lys* lysin, *CM* cysteine /methionine, *Thr* threonin, *Try* tryptophan

**Table 2:** Feed components of the starting and finishing feed

Parameter	value	starting feed	finishing feed
Water	(%)	50.9	50.8
Whey	(%)	25.8	25.7
Commercial feed	(%)	23.3	23.3

The water was taken from a private water supply, which had not been examined for microbiological quality during preceding years. After entering, the pigs received in-feed medication for 10 days with tiamulin hydrogen fumarate (2 mg/kg body weight/day; licensed for prophylactic use in Switzerland) due to a detection of *Brachyspira hyodysenteriae* in previous batches of pigs, although after that pathogen detection a partial sanitation had been conducted on the whole farm. Two days after the antimicrobial treatment, pigs were routinely wormed with flubendazol. The average length of the fattening period was 109 days, and pigs were sent to slaughter with a body weight of 110-120 kg. The daily weight gain was 770-800 g and the feed conversion ratio was 2.7:1. Results at the slaughterhouse were good and the condemnation of carcasses and/or organs very low (1-2% of all livers were condemned).

**Fig. 1:**
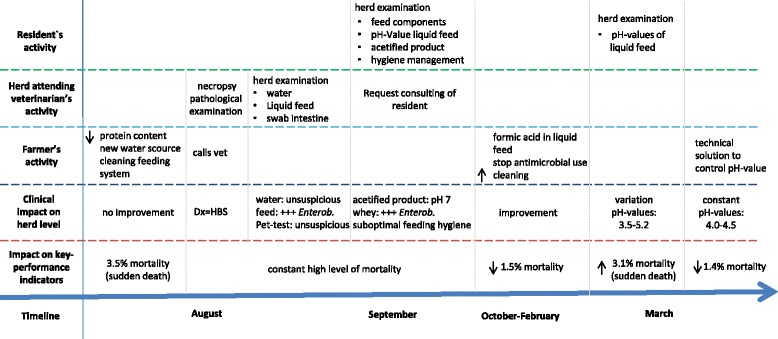
Overview of the chronological succession of this herd health problem. The actitivies of the persons involved and the impact on herd level and herd performance are summarized in a timely manner. Abbreviation: Dx = diagnosis; Enterob. = Enterobacteriaceae; HBS = Haemorrhagic bowel syndrome; +++ = high amount; ↓ decrease; ↑ increase

During the summer of 2015, the farmer noted an increase of the mortality rate from 1.7% to 3.5%, and immediately requested a herd examination. The anamnesis revealed that infrequent sudden deaths of grower pigs between 30 and 60 kg with severe abdominal distension occurring shortly after death had been noted in several pens for several weeks. No changes of the origins of the pigs, housing, water supply or the feeding procedure were reported. An immediate action by the farmer was to reduce the protein content in the feed for growers (no accurate data from the farmer available), to change the water supply from his own spring to municipal water and to improve the cleaning of the liquid feeding system. All these measures did not lead to a significant improvement. A summary of clinical observations including a time course of events is illustrated in Fig. 1.

**Fig. 2:**
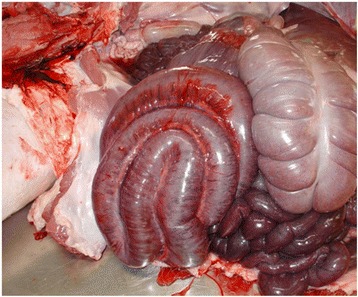
Macroscopic examination of a fattening pig suffering from HBS. A counter clockwise volvulus of 180° of the long axis of the mesentery and a distention of the lumen containing gas and dark red, watery fluid can be seen

Before the herd examination was conducted, the herd veterinarian performed a pathological examination of a fattening pig with a severe abdominal distension that died per-acutely. The necropsy revealed that the small intestine was thin-walled and filled with clotted and unclotted blood. The large intestine contained regular ingesta, but no other pathological findings were observed. For further diagnostics, an aerobic and anaerobic bacterial examination from a swab of the small intestine was performed and revealed a high amount of *Escherichia coli*, *Enterobacter cloacae*, gamma-haemolytic *Streptococci* and *Acinetobacter sp*.. In addition, another dead animal was send to the Institute of Animal Pathology, Vetsuisse Faculty Bern, to confirm the presumptive diagnosis of HBS. The animal had a pronounced distension of the abdomen and the skin showed marked pallor. A counter clockwise torsion of 180° of part of the small intestine around the mesentery root, when viewed from the ventro-caudal aspect of the long axis of the mesentery, was found. The lumen of the intestine was distended with gas and contained dark red, watery fluid (Fig. 2). There was a sharp demarcation between the normal coloured cranial portion of the duodenum and the dark red jejunum and ileum as well as between the ileum and cecum, which was again normally coloured. The post mortem investigation confirmed the diagnosis of HBS. For further diagnostics, a PET-Test from the liquid feed [10], microbiological testing of the water and feed analysis were conducted. No inflation of the balloon within 24 h in the PET-Test could be observed and the microbiological water analysis revealed no abnormalities. The microbiological examination of liquid feed samples from four feeding values revealed a high contamination with *Enterobacteriaceae* in all samples. Further details of the microbiological examination of the liquid feed samples are shown in Table 3.

**Table 3:** Results of microbiological examination from 4 liquid feed samples. Reference values (Kamphues, 2004 [16]) are listed in []. Bold values are beyond the reference values

Sample (feeding value)	*Enterobacteriaceae* (CFU*/g)	*Escherichia coli* (CFU*/g)	Yeast (CFU*/g)
Value 5	**1.400.000** [≤ 10.000]	20 [≤ 100]	15.000 [≤ 100.000]
Value 11	**1.400.000** [≤ 10.000]	70 [≤ 100]	68.000 [≤ 100.000]
Value 34	**> 1.500.000** [≤ 10.000]	10 [≤ 100]	25.000 [≤ 100.000]
Value 39	**> 1.500.000** [≤ 10.000]	20 [≤ 100]	29.000 [≤ 100.000]

* CFU/g: colony-forming unit per gram

After recording the anamnesis, a clinical examination of the fatting pigs was conducted by the first author and all animals on the farm were in good general health condition. The homogeneity within batches was verified and just 1 % of all animals showed growth retardation. Due to the enteric disease problem on herd level, the main emphasis of the herd examination was placed on a thorough examination of the feed and feeding management including the feed storage. The feeding procedure was evaluated in regards to the feeding frequency, potential excitement of pigs during the feeding and the pig place ratio. It was confirmed that the animals showed no significant difference in their behaviour and the animal/feeding place ratio was 1:1. The feed was stored in metal and polyester silos and was checked weekly for contamination, but cleaning was never carried out before a new batch of feed was delivered. The visual examination of all silos showed no abnormalities. The weekly delivery of stabilised whey was stored in a non-chilled metal container. For stabilisation of the whey, the feeding company used their self-established product with different strains of *Lactobacillus*, preserving food salts, enzymes and fungal inhibitor (dosage: 300 g/1000 l whey). For the weekly cleaning of the tank the farmer used a high pressure cleaner and hot water.

Nonetheless, contamination with fat and other material on the surface of the tank were observed during the examination. For further diagnostics, the cleaning management of the feeding system was thoroughly examined. The farmer reported that after every feeding the pipes were flushed with water and fresh water stayed in the whole system until the next feeding. Every 3 months the whole system was cleaned with a 1% sodium hydroxide solution for 30 min. Afterwards the pipes were flushed with feed containing an acidified product to provide a better cleaning effect. In order to prove the efficacy of the acidified product, it was mixed according to the manufacturer’s instructions and the pH-value was measured, revealing values of 7.0 to 7.25. Furthermore, the pH-value of the liquid feed from the mixing vat was evaluated and a value of 5.0 was recorded. Because of the known contamination of the liquid feed, further microbiological examinations of the feeding components (feeding meal, whey, and liquid feed in mixing vat) were conducted.

Before the results of the further examinations were available, it had been recommended that the farmer should control the pH-value and the growth of microorganisms in the liquid feed (origin content) with formic acid (dosage: formic acid 85%; 1.5 ‰ =1.5 l formic acid in 998.5 l whey) in the whey. A range of the pH-value between 4 and 4.5 and frequent recording of the pH-value of the liquid feed were recommended. Furthermore, the producer was asked to stop prophylactic antimicrobial treatment with tiamulin hydrogen fumarate, and instead was advised to improve his cleaning management of the whey tank and also of the liquid feeding system using an acid solution after thorough cleaning. Additionally, necropsies on all sudden deaths were suggested to check the cause of deaths and therefore not overlook a different aetiology for the mortality in the pigs.

Due to these measures the key performance indicators improved significantly and reached the former level.

The tests on the feeding components indicated that the transmission of *Enterobacteriaceace* in the feeding system resulted from contamination of the whey. The details of the microbiological examination of the feed components are described in Table 4.

**Table 4:** Results of microbiological examination of different feed components. Reference values (Kamphues, 2004 [16]) are listed in []. Bold values are beyond the reference values

Sample (component)	Enterobacteriaceace (CFU/g)
Feeding meal	< 10 [≤ 10.000
Whey	**160.000** [≤ 10.000]
Mixing vat	**300.000** [≤ 10.000]

* CFU/g: colony-forming unit per gram

**Fig. 3:**
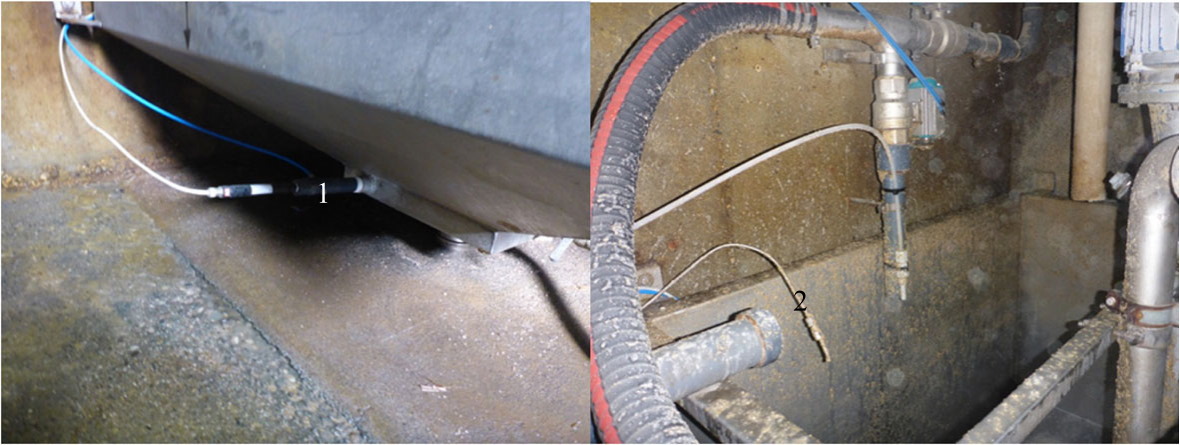
Technical device to measure pH-value in the liquid feed. A pH- value probe (1) permanently measures the pH-value of the feed soup in the mixing vat. An automatic addition of acid from a dosing feeder (2) ensures a constant pH-value in the liquid feed

Four months after the initial herd examination and implementation of control measures, the same clinical manifestation occurred. During a second herd examination, a huge variability of pH-values in the liquid feed ranging from 3.5-5.2 was assessed for different feeding times and were recorded by the farmer. He reported that currently, he was not mixing the formic acid in the whey tank, because the plastic paddles of the mixing machine had been degraded by the formic acid. In order to avoid huge variations of the pH-value in the liquid feed, mixing the acid with the whey using an electric hand drill mixer was recommended. Finally, the farmer implemented a different technical solution to control the pH-value in the liquid feed (Fig. 3). Since implementation of these control measures, the performance characteristics improved significantly and once again reached the former level.

## Discussion

The present report describes a case of HBS in fattening pigs in a system with liquid feeding based on whey. Numerous reports associated whey feeding with occurrence of HBS in pig farms. Pigs fed with whey consume large volumes of liquids and high amounts of easily fermentable components, which predispose for a fermentative imbalance and can cause predisposition for an intestinal volvulus. In addition, it is also necessary to ensure a high level of feed hygiene, because contamination with certain bacteria is also assumed to be a predisposing factor for HBS. Due to the traditional cheese production in Switzerland, an annual amount of 1,352,000 tons of whey accumulate and is mainly used as a protein source in diets for pigs [10]. Whey is often included in these diets because of the high amino acid content and digestibility. However, the high amounts of lactose and sodium as well as the high variability amongst the different batches require a perfect management of whey transport, storage and feeding in order to guarantee a diet of constant quality. In addition, the use of organic acids as preservative is necessary, because whey is highly susceptible to microbial contamination [10].

In the literature, the efficacy of various organic acids in terms of preventing growth of yeast was tested under laboratory conditions [10]. After inoculation with cultures of naturally contaminated whey and the addition of preservatives, the whey samples were incubated during 4 days and yeast loads and gas pressure were assessed. Formic acid proved to be the most efficient whey preservative. The sample admixed with formic acid contained the lowest yeast amount and the smallest fluctuation in pressure. Interestingly, when using propionic acid, the yeast count was not reduced, but the gas pressure frequently decreased below 0 indicating that gas was used by microbes. Therefore, simple PET-tests on farm, which measure gas formation semi quantitatively as an indicator for the presence of yeasts, may yield false negative results [10]. In this case, the PET-test result is in accordance with the microbiological examination showing only a low numbers of yeasts in the liquid feed.

Volvulus of the intestine has been a variable pathological finding in pigs with HBS [2]. Partial or complete volvulus as a common cause of death is indicative for HBS [5]. However, the diagnosis of intestinal torsion is often difficult, because of a potential repositioning of the mesenteric root after death [1, 2, 5]. There are several risk factors described for intestinal volvulus in pigs [1, 2]. Activities such as playing, mounting and fighting can produce an intestinal volvulus. This is even more important when these behaviours occur in parallel with ingesting large volumes of feed and with reduced feeding frequency [1]. The weight of the ingesta is sufficient to rotate the gut. It also seems that genetic changes, such as a longer body in the modern pig breeds, have an impact on the likelihood of intestinal torsions [1]. Furthermore, one study reported a twofold difference in the incidence of HBS between Large White and Landrace pigs [11].

In the present case, the farmer was using antimicrobials as prophylaxis to ensure that another outbreak of dysentery after the partial sanitation did not occur. However, this is not in line with the good standards of antimicrobial use and is counterproductive regarding the aspired reduction of antimicrobials and antimicrobial resistance. Several studies show that even a low, short-term dose of in-feed antimicrobials increases the abundance and diversity of antimicrobial resistance genes, including resistance to antimicrobials not administered [12, 13]. In addition, antimicrobial introduced to the feed provokes a selective pressure that may lead to long lasting changes in livestock commensal microorganisms [14, 15] helping pathogens to multiply in the gastrointestinal tract. This antimicrobial use can cause dysbacteriosis in pigs and therefore could lead to diseases such as HBS.

In the present case, it is likely that a combination of multiple causes such as contamination of the liquid feed with *Enterobacteriaceae*, hygiene problems with the feeding system and dysbacteriosis in the intestinal tract due to antimicrobial treatment, all together led to HBS. Other infectious aetiologies have been described in the literature. It is assumed that HBS arises from overgrowth of bacteria such as *Clostridium perfringens* or *Escherichia coli* in the intestinal tract producing toxins and other damaging compounds. In the present case, no microbiological investigation of faeces samples or intestinal contents was conducted, because of inconclusive discussions in literature and on own experiences. Results of such expensive analysis would most likely not have provided additional information for the case. However, after improving the feed quality by stabilizing the whey with formic acid and increased hygiene of the feeding system combined with stopping of prophylactic antimicrobial treatment, the farmer recorded significantly less mortality. Therefore, a direct association between the different factors and the HBS was concluded.

## Conclusion

In case of HBS a thorough examination of nutritional offering and management practices is necessary to target the specific disease process. Hence, several factors can lead to HBS, and immediate measures against different risk factors are essential to improve herd health and productivity on farm. In the present case report, it is likely that a combination of particular problems such as the contamination of the liquid feed with *Enterobacteriaceace*, the hygiene problem of the feeding system and the chronic dysbacteria of the intestinal tract due to the non-justified routine use of antimicrobials caused HBS. Speculatively, the prophylactic antimicrobial treatment was not only unnecessary, but might even have increased clinical manifestation.

## Abbreviations

%: Percent
CFU/g: Colony-forming unit per gram
g: Gram
HBS: Haemorrhagic bowel syndrome
kg: Kilogram
PET: Polyethylene terephthalate
pH: Potential of Hydrogen
